# Knowledge, attitude and practice of physicians towards herbal remedies in Rasht, north of Iran

**DOI:** 10.1097/MD.0000000000031762

**Published:** 2022-11-25

**Authors:** Soheil Soltanipour, Faeze Keihanian, Amin Saeidinia

**Affiliations:** a Community Medicine Department, Guilan University of Medical Sciences, Rasht, Iran; b Faculty of Medicine, Medicinal Plants Research Center Of Student Basij (MPBG), Guilan University of Medical Sciences, Rasht, Iran; c Cardiology Department, Imam Reza and Ghaem Hospitals, Faculty of Medicine, Mashhad University of Medical Sciences, Mashhad, Iran; d Clinician Scientist of Cardiology, Pharmaceutical Research Division, Booali Research Center, Mashhad University of Medical Sciences, Mashhad, Iran; e Resident of Pediatrics, Faculty of Medicine, Mashhad University of Medical Sciences, Mashhad, Iran.

**Keywords:** attitude, herbal remedies, knowledge, physicians, practice

## Abstract

The World Health Organization has recommended the integration of Complementary and Alternative Medicine (CAM) with modern medicine, as part of the global “health for all” approach. Herbal treatments are the most common and known methods of CAM. Based on the critical role of physicians in true and safe application of these products, this research evaluated knowledge, attitude, and practice (KAP) of physicians in Rasht towards herbal remedies. This was a cross sectional study, performed between April 2017 and May 2019 on all physicians, who worked in Rasht city, northern Iran. A researcher-made questionnaire with 2 main parts, including a demographics section and research questions was used. A total of 142 (38%) individuals responded to the questionnaires. Mean KAP scores were 6.47 ± 6.17, 27.80 ± 3.26, and 5.02 ± 5.54, respectively. In none of these fields, significant differences were seen in participants regarding demographic variables, experience, work place, academic relevance, and field of work (*P* > .05). Physicians of Rasht city, with different work environments and regardless of demographic characteristics, had a positive view towards herbal remedies, without enough knowledge to consult their patients.

## 1. Introduction

CAM is defined by the US National Center for Complementary and Alternative Medicine (NCCAM) as a group of diverse medical and healthcare systems, practices, and products that are not presently considered as part of conventional medicine. CAM is a sub-collection in medical and healthcare systems and has extensive practices and products, which are not defined currently as traditional medicine. There has been an increase in the use of CAM in the last decade, worldwide,^[1–3]^ including Iran.^[4]^ This interest in using CAM is found both in the general population and amongst physicians and health care authorities.^[5]^ Hence, CAM is widely implicated for remedial and hygienic purposes in different nations; 30% to 98% of patients are using some type of CAM.^[6]^ This estimation in industrial countries ranges between one-third and half of the general population.^[6]^ In a study from UK, 33% of individuals used some kind of CAM, this rate was 46% in Australia, 34% in the US, 75% in Belgium, 49% in France, 18% in Netherlands, and 20% to 30% in Germany, which indicates its popularity amongst different societies.^[7]^ In a study from Turkey, 14.5% of 600 patients reported the use of herbal medications, however most of them (92.5%) did not know their side effects.^[8]^ In Saudi Arabia, it was reported that 23.9% of patients, who required assistance from the healthcare system, had a history of traditional medicines.^[9]^

Dissatisfaction with modern medicine, especially patient-physician relationships, and concerns about adverse effects of chemical drugs are reasons for the tendency towards CAM.^[10]^ Difference in socio-economic status of CAM consumers and variability in health problems has led to geographic differences in the level of CAM use.^[11,12]^ However, herbal medicine is the most common method used in CAM.^[3,13–15]^ It seems that herbal remedies are majorly preferred for chronic health problems, which decrease quality of life.^[11,16]^ The WHO has suggested combination of CAM with the modern medical system as part of the world strategy of “health for all.”^[11]^ According to the increasing interest in the use of CAM, physicians should prepare for indications, limitations, and complications of these remedies. They should look for suitable choices of CAM, where there is no compliance to use modern medicine prescriptions.^[12]^ Various investigations have evaluated the KAP of physicians towards CAM. However, such studies have limitations and methodological problems, such as small sample size or low response rate.^[17]^ People’s low amount of knowledge about herbal medicine or medicinal plants has led to uncontrolled utilization of these products.^[11]^ In this study, the researchers assessed KAP of physicians of Rasht city, Guilan, north of Iran, towards medicinal plants and herbal medicine.

## 2. Methods

This was a cross sectional study, which was performed between April 2017 and May 2019.

### 2.1. Study population

The study population was all physicians, who worked in Rasht city. Sampling was done by the stratified random sampling method. The researchers obtained a list of physicians from the treatment-assistance unit of Guilan University of Medical Sciences (GUMS), as the sampling frame. The physicians were categorized in 3 subsets: general physicians, specialists, and subspecialists. By using http://www.randomizer.org, random numbers were extracted and used for selection of each subset. According to previous studies about the participation rate of physicians,^[18,19]^ estimated sample size, with OpenEpi software version 2 2008 (Atlanta, GA) was considered 600 subjects (confidence interval [CI]: 99%, *α* = 0.01). For each strata, regarding to population size, sample size were estimated. Population sizes of general physician, specialists and subspecialists were 39%, 52% and 9%, respectively. Final sample size for study calculated for general physicians, specialists and subspecialists as 152, 302 and 35 persons.

### 2.2. Interview instrument

This study used a researcher-made questionnaire with 2 parts, including demographic questions and research questions. The question part included 6 questions for knowledge measurement (score 0–30), 8 questions for attitude measurement (scores between 8 and 40; each question was answered on a Likert scale: highly agree, agree, have no comment, disagree, and highly disagree), and 6 questions for practice measurement (score between 0 and 25). Attitude part has 1 non-Likert question and practice part has 4 non-Likert questions those were reported separately but not involved in scoring (supplementary 1, Supplemental Digital Content, http://links.lww.com/MD/H922).

### 2.3. Knowledge measurement

The physicians gave scores for each answer. This means that for each true answer, they gave 1 point and for each wrong answer or no answer, they gave 0 points. For 6 questions and maximum of 5 answers, the maximum of knowledge was 30 and the minimum was zero. The score of knowledge was the sum scores of these 6 questions, and a higher sum of answers indicated greater knowledge.

### 2.4. Attitude measurement

The 8 questions in the attitude part were scored on a Likert scale, except 1 question, which was not Likert-based. The “highly agree” option was given a score of 5 points, “agree” had 4 points, “no comment” had 3 points, “disagree” had 2 points, and “highly disagree” had 1 point. If the sum of answers was higher, it reflected that the physician had a more positive view towards herbal medicine and if it was lower, it meant the physician had a more negative view towards herbal medicines.

### 2.5. Practice measurement

Regarding questions on practice, the participants also scored their answers as 1 or 0. This means for each true answer, they gave a score of 1 and for each wrong answer or no answer, they gave a score of 0. For 5 questions and maximum of 5 answers, the maximum of knowledge was 25 and the minimum was 0. The score of knowledge was the sum of the scores for the 5 questions, and if the sum of the answers was higher, greater practice was indicated.

Herbal remedies were defined as listed by the Iranian National Pharmacopeia of food and drug association of Iranian Ministry of Health. The reliability of the questionnaire was evaluated and approved by an expert panel and its stability was established by a pilot study on 30 physicians, and its Cronbach alpha was determined as 0.81. Questionnaires were completed by an interview with educated researchers in Rasht and at the clinics of the physicians. The researchers attended the clinic of physicians and if the physician agreed to participate in the research, they were asked to respond to the questionnaire. If physicians did not agree to participate in the study, the researchers asked them to express their reason and recorded their responses.

### 2.6. Ethics

This study was approved by the ethics committee of research and technology of Guilan University of Medical Sciences (GUMS) with code number 90060008, and the researchers followed the principles of the ethical committee of the university. The names of all participants were kept confidential.

### 2.7. Statistics

Data were entered in the SPSS software version 16.0 (SPSS Inc. Chicago, Illinois) and reported by descriptive indexes, including means, standard deviations, and frequencies. Mann–Whitney *U*, Chi square, and Fisher exact tests were used for the analysis.

## 3. Results

### 3.1. Demographics

Of 650 distributed questionnaires, 142 physicians participated in the study and the response rate was 21.84%. From all subjects (650 physicians), 432 were male (66.4%) and 218 were female (33.6%) with mean age of 46.1 ± 10.8 years old. Sixty-six percent (432 cases) were general physicians, 31% (202 cases) were specialists, and the rest (16, 2.5%) were subspecialists. Overall, 118 individuals worked at educational university centers. Furthermore, 556 (85.5%) worked at governmental centers. All demographic data are listed in Table 1. Amongst subjects that participated in the study, the mean age was 52.11 ± 13.47 years old. One hundred four of them (73.2%) were male and 38 were female (26.7%). Other features are listed in Table 1. Table 2 indicates the reason for lack of participation. They had to choose only 1 reason for their lack of participation.

**Table 1:** Demographic data of all physicians requested to fill the questionnaire.

Characteristics	Participation			*P* value
	Yes (n = 142)	No (n = 508)	Total (n = 650)	
Age (mean ± S.D) yrs	52.11 ± 13.47	44.10 ± 48.03	46.1 ± 10.80	0.001^*^
Gender (N, %)	Male (104, 73.2) Female (38, 26.7)	Male (328, 64.6) Female (180, 35.4)	Male (432, 66.4) Female (218, 33.6)	.111^**^
Experience (mean ± S.D) yrs	22.11 ± 23.41	14.80 ± 16.52	22.23 ± 11.41	.001^*^
Work place (N, %)	Governmental (60, 42.3) Private (82, 57.7)	Governmental (496, 97.6) Private (12, 2.4)	Governmental (556, 85.6) Private (94, 14.4)	.001^**^
Academic relevance^¶^ (N, %)	Yes (84, 59.2) No (58, 40.8)	Yes (34, 6.7) No (474, 93.3)	Yes (118, 18.2) No (532, 81.8)	.007^**^
Level of working (N, %)	General (54, 38) Specialist (80, 56.3) Subspecialist (8, 5.6)	General (378, 74.4) Specialist (122, 24) Subspecialist (8, 1.6)	General (432, 66.4) Specialist (202, 31.1) Subspecialist (16, 2.5)	.001^**^

* Mann–Whitney *U* test.

** Chi-square test.

^*¶*^ Work in educational centers.

**Table 2:** Causes of non-tendency to participation.

Cause	Number	Percent
Non-prescription of herbal remedies	20	3.9
Not believe herbal remedies	158	31.1
Unknown complications of herbal remedies	6	1.2
No enough scientific evidence for prescription	18	3.5
None of knowledge about it	10	2
Non-efficiency of herbal remedies	4	0.8
Difficult name of herbal remedies	2	0.4
Non-applicability of herbal remedies in my field of work	8	1.6
No time for interview	282	55.5
Total	508	100

### 3.2. Knowledge

The mean score of knowledge in participants was 6.47 ± 6.17 (mean: 0, max: 25). Mann–Whitney *U* test indicated that there was no significant association between knowledge score and gender (mean rank for males: 34.26, mean rank for females: 41.11; *P* = .217), academic relevance (mean rank for academics: 26.19 and mean rank for non-academics: 38.20; *P* = .054), job level (mean rank for general practice: 38.20, mean rank for specialist/subspecialist: 26.19; *P* = .054), and place of work (mean rank for the governmental sector: 40.66, mean rank for the private sector: 34.65; *P* = .298). Spearman rho showed that there was no significant correlation between score of knowledge, age (*P* = .581), and experience (*P* = .815).

### 3.3. Attitude

The mean score of attitude of participants was 27.80 ± 3.26 (minimum: 8 and maximum: 40). Mann–Whitney *U* test indicated that there was no significant difference between the attitude score and gender (mean rank for males: 34.75 and mean rank for females: 39.67; *P* = .378), academic relevance (mean rank for academics: 27.46 and mean rank for non-academics: 37.91; *P* = .096), job level (mean rank for general practice: 39.98 and mean rank for specialist/subspecialist: 33.56; *P* = .198), and place of work (mean rank for governmental sector: 43.69 and mean rank for private sector: 33.76; *P* = .087). Spearman rho showed that there was no significant correlation between score of attitude, age (*P* = .508), and experience (*P* = .449). Fourteen cases (9.9%) mentioned that they did not prescribe herbal drugs because of “lack of effectiveness”; 24 (16.9%) did not prescribe these drugs because of “not being scientific” and 12 (8.5%) did not prescribe these drugs because of other causes. Ninety-two participants did not respond to this question (64.8%).

### 3.4. Practice

The mean score of practice in participants was 5.02 ± 5.54 (minimum: 0 and maximum: 25). Mann–Whitney *U* test indicated that there was no significant difference between practice score and gender (mean rank for males: 34.29 and mean rank for females: 41.03; *P* = .216), academic relevance (mean rank for academics: 28.27 and mean rank for non-academics: 37.73; *P* = .122), job level (mean rank for general practice: 39.98 and mean rank for specialist/subspecialist: 33.56; *P* = .198), and place of work (mean rank for governmental sector: 43.69 and mean rank for private sector: 33.76; *P* = .087). Spearman rho showed that there was no significant correlation between score of practice, age (*P* = .699), and experience (*P* = .627). Table 3 shows the choices of participants in the second part of the practice field.

**Table 3:** Distribution of answers of participants and their participation rate in the second part of practice questions.

Questions	Choices	Answer frequency (N, %)	Answer rate (%)
On average, in every 20 patient visits, how many times do you prescribe at least 1 herbal remedy?	1–4	80, 56.3	67.6
	5–8	8, 5.6	
	9–12	8, 5.6	
For which of these problems do you prescribe herbal remedies?	Chronic disorders (asthma, osteoarthritis, etc.)	22, 15.5	25.3
	When common drugs have no effect	32, 22.5	29.5
	Diseases which have no certain treatment	16, 11.3	22.5
Do you give any information to your patients about role of herbal remedies in health?	Yes	82, 57.7	71.8
	No	20, 14.1	
Do you prescribe herbal remedies for your family?	Yes	82, 57.7	71.8
	No	20, 14.1	

## 4. Discussion

Studying KAP of a population can significantly help policy makers to make the best decisions regarding relevant issues.^[20]^ The use of CAM and herbal medicine has become increasingly popular, worldwide.^[14,21,22]^ In developed countries, the accelerating improvement in the health system has been due to demodulation of new active chemical constitutes from herbal medicine and use of traditional herbal medicine for modern healthcare improvements. Therefore, knowledge of the western-trained system towards herbal medicine, as a CAM choice, is very low.^[23]^

Lack of participation had a rate of 78.16%, which was due to lack of time (55.5%) and disbelief in herbal remedies (31.1%). The response rate in similar studies was somewhat in line with the current investigation: 38.04%,^[24]^ 41%,^[25]^ 51.2%,^[27^ 66.7%,^[27]^ 70%,^[10]^ and 88%.^[28]^ This indicates that lack of participation was meaningful. However, in contrast to most other studies, physicians were asked about their reason for lack of participation. This low participation and disbelief in herbal remedies could be related to curriculum of general medicine, in which there is no data regarding CAM. Therefore, lack of adequate training during undergraduate and postgraduate years of study may be the reason for physician’s inability to undertake these tasks confidently.^[29]^

The level of knowledge towards herbal remedies was very low in the current study (6.47/25). This low knowledge was not related to gender, experience, work place, job level, and environment of medical practice (*P* > .05). Inversely, in another study, it was shown that gender (males) and experience were significantly related to higher knowledge, despite lower knowledge in all participating physicians.^[27]^ This low knowledge in physicians was reported previously in other studies. Ventola et al^[30]^ revealed that 46.6% of their study population had weak or very weak knowledge in this field. Senobar-Tahani et al^[31]^ showed that 22.2% of their study population had moderate knowledge and 23.5% had low knowledge towards herbal remedies. In other studies, weak knowledge was reported; Alrashidi et al^[28]^ reported that 62.5% of their cases had weak knowledge; Levine and Xu^[32]^ and Clement^[23]^ indicated that the knowledge of physicians was poor. This can be because of

1. lack of medical education on CAM, especially herbal remedies;
2. lack of attention to this topic in educational courses;
3. lack of a complete and comprehensive national guideline for physicians in different parts of the world in order to prescribe herbal medicines and understand the benefits or adverse effects;
4. existence of nonacademic individuals, who work at herbal stores without any supervision, and, who deny modern medicine.

This study showed that the participants had a positive view and attitude towards herbal medicine (27.8/40). This finding is in line with Furlow et al’s study, in which 41.2% of physicians believed that herbal medicine could be moderately to highly effective.^[25]^ The results were similar to some other studies that evaluated CAM or herbal medicine in different physician populations: Kemper and O’Connor^[33]^ indicated that 66% of pediatricians had a positive view towards CAM in the US. According to Junaid et al,^[34]^ in Pakistan, 43% of general practitioners stated that herbal medicine was the most effective approach in CAM. Ghia and Jha studied physicians working at Indian hospitals,^[35]^ Levine and Xu investigated residents and clinical clerks in Canada,^[32]^ Hilal and Hilal studied different physicians in Bahrain,^[27]^ and Senobar-Tahani et al evaluated physicians in Iran.^[31]^ The positive attitude towards using herbal remedies could be related to patients’ inquiries about herbal medicine, widespread advertisements in this field, advances in herbal remedies, introduction of new drugs in reference books from these products, and finally growing number of clinical trials and meta-analysis on the use of herbal medicines in different disorders.

Regarding utilization, the mean score obtained in the current study was low (5.02/20) and it was not significantly related to an underlying factor. In the current study, most physicians used herbal medicine 1 to 4 times, in every 20 patient visits. The most common etiology for using herbal medicine in the current study was related to chronic disorders, non-responding conventional remedies, and lack of treatment for a disorder, respectively. In another study, herbal medicine was used for preventing disorders, alleviating symptoms, immune system alimentation, increasing energy, and treatment of non-treatable disorders.^[26]^ Furlow et al reported that 61.4% of physicians in their study utilized herbal remedies as part of CAM for their patients.^[25]^ Some other studies, such as that of Levine and Xu,^[32]^ Dougherty et al,^[26]^ and Clement et al,^[23]^ also explored high utilization of herbal medicine by their participant physicians. In other studies, French and Danish physicians were prescribed at least 1 herbal medicine prescription in 31% and 12% of visits, respectively.^[17]^ Lower score of utilization in the current study was predictable, according to lower knowledge score. However, high prescription number per visit could indicate greater knowledge. KAP study models are being done for changing the views of participants towards a problem and can be used as a preface for this alteration.^[36]^ Doctors should be aware of the potential interactions between traditional medicines, herbal medicines, and cultural context, and use herbal remedies as appropriate in their field of work.^[8]^ Lower prescription rate of physicians of herbal medicine is due to several reasons, including

1. lower experience of physicians trained in conventional medicine towards herbal medicine;
2. lower knowledge of physicians in this field;
3. lack of national evidence-based guidelines for herbal medicines in different geographic areas, such as Physician Desk Reference (PDR) for herbal medicine book prepared by the WHO;
4. lack of availability of physicians for international guidelines, like the German Commission E,^[37]^ physician desk reference (PDR),^[38]^ European Scientific Cooperative On Phytotherapy (ESCOP),^[39]^ as a complementary reference for continuous medical education courses.

Our study had some limitations: we did not have access to physicians in hospitals well, because their environment for interview was not suitable. Another was the low rate of participation in the study, because of some negative view toward herbal medicines and its integration with superstitions in people’s ideas.

## 5. Conclusion

The current results demonstrated that physicians of Rasht city, in different work places and regardless of demographic characteristics, had a positive view towards herbal remedies without enough knowledge in this field for prescribing these medicines. There is a gap between positive attitude and knowledge of physicians and this is a chance for healthcare system policy makers to use this direction for future programming of education courses for medical practitioners. Well-informed practitioners can make better communication with their patients and inform them in the field of herbal medicine. Considering the current results, making a national evidence-based guideline or booklet, according to the Iranian traditional medicine and its herbal remedies, which would provide information about common names, traditional utilization options, therapeutic indications, scientific names, known interactions with conventional drugs, pharmacological features, and chemical compounds responsible for their effects could be a good source for physicians, and can be taught in the curriculum of general physicians and in continuous medical education programs for current physicians in the healthcare system.

## Acknowledgments

The authors thank Guilan University of Medical Sciences and Medicinal Plants Research Center of Student Basij (MPBG), for their support. The authors thank Dr Masoume Shafiei and Dr Zahra Akbarian for assisting with the questionnaire validation.

## Author contributions

**Conceptualization:** Soheil Soltanipour, Amin Saeidinia.

**Data curation:** Soheil Soltanipour.

**Formal analysis:** Soheil Soltanipour.

**Investigation:** Soheil Soltanipour, Faeze Keihanian, Amin Saeidinia.

**Methodology:** Soheil Soltanipour, Faeze Keihanian, Amin Saeidinia.

**Project administration:** Faeze Keihanian.

**Supervision:** Faeze Keihanian.

**Writing – original draft:** Faeze Keihanian, Amin Saeidinia.

**Writing – review & editing:** Soheil Soltanipour, Faeze Keihanian, Amin Saeidinia.

## Supplementary Material
