# Supplementary material for: Knowledge, attitude and practice of physicians towards herbal remedies in Rasht, north of Iran
Source: Medicine (Baltimore). 2022 Nov 25;101(47):e31762. doi: 10.1097/MD.0000000000031762 (PMC9704890; doi:10.1097/MD.0000000000031762)
Supplement: Supplementary file 1 [file medi-101-e31762-s001.pdf]

Appendix 1- Questionnaire questions

Knowledge questions	
1. Can you name any herbal medicine? Please name ….	
2. Can you please number any clinical use for each named herbal medicine?	
3. Can you please say a contraindication for each named herbal medicine?	
4. Can you please say a drug interaction for each named herbal medicine?	
5. Can you please say an adverse reaction related to each named herbal medicine?	
6. Could you please tell us name of some of herbal medicine pharmacopeia?	
Attitude Questions	*(Likert scale except number 7 question: Highly Agree, agree, no comment, disagree, highly disagree)*
1. I am interested in herbal medicine.	
2. Herbal medicine and its products can be applicate clinically separately.	
3. Herbal medicine and its products along with conventional medicine can be used.	
4. Effect of herbal medicine is usually because of its placebo effect.	
5. Herbal medicine should be research and evaluate more by universities.	
6. If an herbal medicine has an equal effect with chemical drug, I choose herbal medicine.	
7. I have not prescribed herbal medicines because: a) non-hygienic standard preparation, b) non-efficacy, c) not evidence-based, d) adverse effects of them, e) other (please name ….)	
8. What is your opinion for putting herbal medicine education in continuous medical education programs?	
9. What is your opinion for putting herbal medicine education in general practitioner training curriculum?	
Practice questions	
1. Did you ever prescribe herbal medicine? (If your answer is positive please answer left questions).	
2. Please name some herbal medicine prescribed.	
3. Please say a clinical use for each named herbal medicine.	
4. Please say a contraindication for each named herbal medicine.	
5. Please say a chemical drug for each named herbal medicine, that make drug interaction.	
6. Please say an adverse drug reaction for each named herbal medicine.	
7. On average, in every 20 patient visits, how many times do you prescribe at least one herbal remedy?	
8. For which of these problems do you prescribe herbal remedies?	
9. Do you give any information to your patients about role of herbal remedies in health?	
10. Do you prescribe herbal remedies for your family?	
